# #infertility in Brazil: an analysis of authorship and post content on Instagram

**DOI:** 10.5935/1518-0557.20240081

**Published:** 2024

**Authors:** Márcia Mendonca Carneiro, Caio Nobuyoshi Koga, Marcela Chagas Lima Mussi, Pollyanna Faria Fradico, Marisa Mendonça Carneiro

**Affiliations:** 1 Departamento de Ginecologia e Obstetrícia da Faculdade de Medicina da Universidade Federal de Minas Gerais, Belo Horizonte, MG, Brazil; 2 Faculdade de Medicina da Universidade Federal de Minas Gerais, Belo Horizonte, MG, Brazil; 3 Faculdade de Letras da Universidade Federal de Minas Gerais, Belo Horizonte, MG, Brazil

**Keywords:** infertility, fertility, social media, assisted reproduction, IVF, oncofertility

## Abstract

**Objective:**

To use hashtag and content analysis to determine the prevalence, authorship, and types of fertility-related and oncofertility information shared on Instagram in Brazil.

**Methods:**

Eleven fertility-related hashtags were chosen according to https://www.tagsfinder.com/: #infertility, #ivf, #endometriosis, #tryingotconceive, #maternity, #humanreproduction, #pregnancy, #invitrofertilization, #assistedreproduction, #pregnant, #difficulttogetpregnant. For oncofertility, the hashtags were #cancerandpregnancy, #oocytecryopreservation), #oncofertility, and #fertilitypreservation. Posts receiving over 20 comments in March 2021 were analyzed for topic and content. They were divided into six categories (educational, patient experience, outreach, emotional, social support, and coping mechanisms) before being analyzed by three authors. Authorship was divided into healthcare professionals (HCP) and lay people (LP). Posts not written in Brazilian Portuguese, videos, reposts of another account, and commercial posts were excluded.

**Results:**

A total of 220 posts with fertility-related terms were analyzed. HCP accounted for 52% (n=114) and LP for 48% (n=106). Most HCP posts were made by medical doctors (n=78; 68.4%) and fertility clinics (n=27; 23.6%). Authorship analysis (HCP *vs*. LP) for each hashtag was as follows: #infertility (35% *vs*. 65%), #ivf (35% *vs*. 65%), #endometriosis (45% *vs*. 55%), #tryingtoconceive (65% *vs*. 35%), #maternity (LP 100%), #humanreproduction (20% *vs*. 80%), #pregnancy (95% *vs*. 5%), #invitrofertilization (50% *vs*. 50%), #assistedreproduction (5% *vs*. 95%), #pregnant (85% *vs*. 15%), #difficulttogetpregnant (5% *vs*. 95%). The category breakdown was as follows: 39% educational, 19% emotional, 16% social support, 13% patient experience, 9% coping, and 4% outreach. A total of 98 posts with #oncofertility were retrieved. Most (77.5%; n=62) were written by HCP. They fell into the following categories: 55% educational, 15% patient experience, 12.5% emotional, 11.2% support, and 3.2% coping.

**Conclusions:**

Both HCP and LP write about major fertility topics on Instagram. Oncofertility appears to gain less attention. In Brazil, educational content, followed by emotional content, prevails on Instagram.

## INTRODUCTION

Infertility affects 1 in 6 adults globally, according to a document released by the World Health Organization (WHO) (WHO, 2023). Numbers are higher in the Americas (20%) and the Western Pacific (23.2%). The American Society for Reproductive Medicine (ASRM) has updated the definition of infertility to be more inclusive and embrace the diverse population that may need medically assisted reproduction (Practice Committee of the ASRM, 2023). Unfortunately, access to diagnosis and treatment is a significant issue worldwide, especially in lowand middle-income countries (Njagi *et al*., 2023). Many people turn to online resources to obtain information. It is no secret that the use of social media as a source of health-related information has escalated in recent years. Reasons people search social media for information on healthcare topics may vary but include finding out about a specific disease or a medical doctor/hospital, obtaining guidance or a second opinion, and connecting with others facing the same issue (Farsi *et al*., 2022).

The way people face their infertility journey varies, as the road to conception and family building can be challenging. Nearly 50% of infertile couples never consider treatment, and almost 25% are unable to carry on due to the emotional distress involved (Domar *et al*., 2021). Moreover, little financial and emotional support is available to infertile couples. In this hostile setting, social media platforms surface as a source of information and emotional support (Sormunen *et al*., 2020; Farsi *et al*., 2022). Social media may be the only source of information for many people as answers and specific diagnoses are readily available. However, prompt access to unverified information may pose relevant issues and lead to misconceptions. Anyone can post about almost anything on social media, and studies have indicated that caution is warranted given the proliferation of misinformation in posts on relevant public health topics such as smoking, drugs, and vaccines (Suarez-Lledo & Alvarez-Galvez, 2021).

Oncofertility is a new interdisciplinary area in fertility care that addresses the issue of gonadotoxicity associated with cancer treatments and aims at preserving reproductive potential (Sehring *et al*., 2021). Although known for years, cancer treatment-related fertility issues remain overlooked and, at times, inadequately addressed. According to the REDLARA 2019 report, only 5.7% of the 7,990 fertility preservation cycles initiated in 2019 were due to cancer-related factors (Zegers-Hochschild *et al*., 2022). Reasons for such low usage of oncological fertility preservation may include a lack of awareness at all levels of care (professionals and organizations), patients focusing on overcoming and surviving cancer, and the costs involved (van den Berg *et al*., 2019). As cancer cases and survivorship increase and women postpone maternity, the need for education on cancer prevention and oncological fertility preservation becomes evident (Sehring *et al*., 2021).

Instagram is a free online social media platform with about 122,9 million users in Brazil (42.39% males and 57.61% females) (Bianchi, 2024). Brazil is the fifth-largest social media market in the world and the number two in the American continent (CR Team, 2023). Despite the large audience and the educational potential Instagram presents, the use of Instagram in fertility/infertility care needs to be investigated appropriately. Studies in the United States reveal most posts are authored by patients who want to share their infertility journey. Physicians and medical societies have had little participation (Blakemore *et al*., 2020; Peyser *et al*., 2021). We found that no studies have been published on the use of Instagram in fertility care in Brazil. As millions of Brazilians use Instagram as a significant source of information, we aimed to determine the prevalence, authorship, and types of fertility-related information, including oncofertility, shared on Instagram in Brazil using hashtag and content analysis. Secondary endpoints included the analysis of post content according to author type (healthcare professional versus lay people) and the identification of topics in these interactions.

## MATERIAL AND METHODS

Eleven hashtags consisting of fertility terms commonly used on Instagram were chosen based on a list suggested by https://www.tagsfinder.com/ (accessed on February 15, 2021). The list included #infertility, #ivf, #endometriosis, #tryingtoconceive, #maternity, #humanreproduction, #pregnancy, #invitrofertilization, #assistedreproduction, #pregnant, and #difficulttogetpregnant. The corresponding hashtags in Brazilian Portuguese were: #infertilidade, #fiv #endometriose, #tentante, #maternidade, #reproducaohumana, #gravidez #fertilizacaonvitro, #reproducaoassistida #grávida, #dificuldadeparaengravidar.

For oncofertility, the following hashtags were chosen: #cancerandpregnancy (#canceregravidez), #oocytecryopreservation (#congelamentodeovulos), #oncofertility (#oncofertilidade) and #fertilitypreservation (#preservacaodefertilidade).

These hashtags were chosen for their potential use by individuals seeking fertility information. Our Instagram hashtag search and analysis methods were based on previous studies that considered the most common hashtags posted by fertility social media influencers (Blakemore *et al*., 2020; Peyser *et al*., 2021).

A new Instagram account (@resmedgob) was created to avoid potential platform algorithm biases and evaluate the hashtags of interest. Three study investigators (CK, MCM, and PF) performed content analyses, which were double-checked by a fourth (MMC).

In March 2021, the most recent 20 posts containing the abovementioned hashtags were analyzed. Posts that received over 20 comments were analyzed to determine the topics that prevailed in those interactions.

We adopted Peyser’s authorship categories and a combination of Peyser’s and Perone’s categories for content analysis (Perone *et al*., 2020; Peyser *et al*., 2021). We excluded the advertisement and research categories since we did not include commercial posts. We also excluded the personal category and added Perone’s categories of emotion, social support, and coping mechanisms, which are more specific and might help understand the spectrum of emotions affecting patients dealing with infertility. Posts not written in Brazilian Portuguese, video posts, reposts of other accounts, and posts selling products and services were also excluded. The following categories were used in content analysis: educational, patient experience, outreach, emotion, social support, and coping. Three authors analyzed posts falling into these categories, and a fourth double-checked them (Marcia M Carneiro), as described above. Categorization doubts were sorted out among authors.

The definitions of each category were based on Peyser *et al*. (2021). Educational posts included those aimed at providing education or information on fertility themes such as infertility diagnosis or workup. Outreach posts included those aimed at engaging users by encouraging comments or asking questions such as “Leave a comment if you have had a low response to medication.” Patient experience included accounts of an individual’s journey during treatment, and social support revolved around interactions with individuals in IVF journeys. The coping category discussed strategies to deal with treatment stress and difficulties, while emotion referred to posts in which the emotional aspects of fertility were addressed.

Authorship was divided into healthcare professionals (HCP) and lay people (LP). In the HCP category, we included physicians, other healthcare workers (e.g., nurses, psychologists, and pharmacists), and professional societies (Brazilian Society of Assisted Reproduction, for example).

Text, hashtags, and relevant comments were extracted from each post. Relevant post screenshots were taken, and the links to all posts were individually transcribed onto our database. The frequencies of occurrence of each topic and authorship were calculated. Statistical analyses were restricted to descriptive of the frequency of each outcome. Three authors categorized the posts. In cases of disagreement, a fourth author was involved in defining the category to which the post belonged.

No ethical approval was required as we used only publicly available information, and our evaluation did not disclose information that might allow the identification of the posts’ authors (Brazil, Ministério da Saúde. Conselho Nacional de Saúde, 2016).

## RESULTS

Our search yielded 220 posts. Healthcare professionals were responsible for most posts (Table 1) but were closely followed by laypeople.

**Table 1:** Authorship:#infertility HCP (healthcare professional) LP (laypeople).

Authorship	Nº	%
HCP	114	52%
LP	106	48%

Most HCPs posting infertility-related hashtags were medical doctors (n=78; 68.4%) and fertility clinics (n=27; 23.6%). When analyzed for authorship, #maternity was 100% used by LP, while #pregnant, #pregnancy, and #trying to conceive were primarily used by HCP. Interestingly, the majority of posts on technical topics such as infertility, IVF, human reproduction, and assisted reproduction were authored by laypeople.

A total of 98 posts on #oncofertility were retrieved for analysis. Most (77.5%; n=62) were written by HCPs, the majority of whom were medical doctors (n=52; 65.0%), followed by fertility clinics (n=7; 8.75%), psychologists (n=2; 2.5%), and one biomedical scientist (1.25%).

Technical terms such as oncofertility, fertility preservation, and oocyte freezing were almost exclusively used by HCPs (Table 2). Fertility-related hashtags were placed in the following categories: 39% in educational, 19% in emotional, 16% in social support, 13% in patient experience, 9% in coping, and 4% in outreach (Table 3). Oncofertility-related posts were categorized as follows: 55% in educational, 15% in patient experience, 12.5% in emotional, 11.2% in patient support, and 3.2% in coping. Content analysis (Table 3) reveals most posts were in the educational (39%) category, followed by emotional and social support.

**Table 2:** Hashtag subject analysis according to authorship: HCP (healthcare professional) LP (laypeople).

Hashtag	HCP	LP
#infertility	35%	65%
#ivf	35%	65%
#endometriosis	45%	55%
#tryingtoconceive(ttc)	65%	35%
#maternity	0%	100%
#humanreproduction	20%	80%
#pregnancy	95%	5%
#invitrofertilization (IVF)	50%	50%
#assistedreproduction	5%	95%
#pregnant	85%	15%
#difficulttogetpregnant	5%	95%
#cancerandpregnancy	30%	70%
#oncofertilty	90%	10%
#fertilitypreservation	100%	0
#oocytefreezing	90%	10%

**Table 3:** #infertility post content analysis.

Content	%
Educational	39%
Emotional	19%
Social support	16%
Patient experience	13%
Coping	9%
Outreach	4%

Authors agreed on the categorization of most posts (58%). As coping strategies may be used in response to mixed emotions throughout the infertility journey and entail social support, in some instances, it was challenging to decide on the main subject of the post to categorize it properly. In such cases, doubts were sorted out among the authors, considering the definitions set out by Peyser’s and Perone’s categories for content analysis (Perone *et al*., 2020; Peyser *et al*., 2021).

## DISCUSSION

Our results revealed that infertility is a topic that attracts attention on Instagram in Brazil. Both healthcare professionals and laypeople have written posts on the subject. Medical doctors and fertility clinics appear to dominate the Brazilian social media landscape as far as hashtags involving infertility and oncofertility are concerned. Educational posts are the most common, but emotional and social support are also relevant categories. It is also interesting to see that lay people produce the majority of posts using technical terms such as IVF, human reproduction, and assisted reproduction. In oncofertility, the participation of laypeople is dramatically reduced, and healthcare professionals write most posts on the subject.

Brazil is a critical player in the social media scene. Brazilian IVF clinics are mindful of the relevance of social media, and most are present on Facebook (79.5%) and Instagram (75.8%) (Carneiro *et al*., 2023). This is the first study to look at the use of social media in infertility/oncofertility in Brazil. Infertility affects 1 in 6 people globally. Many with the condition seek information and knowledge on social media platforms such as Instagram, where post content and authorship are highly relevant. In Brazil and Latin America, access to proper fertility/oncofertility care sadly remains the privilege of a few due to the high costs involved, the lack of health insurance coverage, and the scarcity of publicly funded fertility clinics (Machin *et al*., 2020; Zegers-Hochschild *et al*., 2022). Healthcare professionals must be made aware of the need to improve patient education (Zegers-Hochschild *et al*., 2022).

A few papers have been published on the role of social media in the fertility journey, but they are restricted to authorship evaluation. Content quality needs to be thoroughly analyzed and is a matter of concern. Our results on authorship show that laypeople account for 48% of the posts on infertility. Laypeople had publications on every topic of interest, except for #pregnancy, #pregnant, and #tryingtoconceive. The majority of posts on technical terms such as infertility, IVF, assisted reproduction, and human reproduction were authored by laypeople. It is possible that healthcare professionals chose non-technical and non-scientific terms to facilitate comprehension. This means people are reading information on in vitro fertilization, endometriosis, and assisted reproduction from sources that may not be evidence-based or have the knowledge to explain complex treatments adequately. People looking for information on conception are more likely to seek help online before talking to a medical doctor, which means they might have access to poor-quality information first. Blakemore *et al*. (2020) found the most frequent fertility content on Instagram was personal stories (31.7%) and inspiration or support (23.7%), while education was a significant topic in our study. The authors found that most accounts in the fertility-related social media landscape belonged to patients, inspirational accounts, and support groups, all of which were more influential than physicians, medical societies, or fertility clinics with social media accounts. In Brazil, healthcare professionals and laypeople share almost evenly the authorship of posts on Instagram. Therefore, Brazilian fertility specialists and medical societies should attempt to increase their involvement in social media to educate, provide support, and increase awareness of patients.

The dissemination of misinformation on social media platforms is a primary concern in several areas of healthcare. Despite the widespread availability of information online and on social media, knowledge gaps remain related to relevant aspects of human reproduction, including the correct “fertile window” (Hammarberg *et al*., 2017). There is an urgent need to grasp the dynamics of social media and develop ways to counteract misinformation on healthcare. Misinformation may be particularly hazardous in fertility care when the web and social media are the only sources of information available to potential patients for whom access to sophisticated procedures relies on their ability to afford treatment (Machin *et al*., 2020). The Brazilian Instagram scene in healthcare needs to be better understood, and contents more closely probed for quality. Unfortunately, it is not difficult to find accounts selling miracle treatments and pills that supposedly improve pregnancy rates in substitution for IVF, which may create false hopes and add to the burden these patients face on their road to fertility.

Oncofertility is not a popular theme on Brazilian Instagram, which perhaps reflects the low use of fertility preservation in individuals with cancer in Latin America, as indicated by the 2019 REDLARA report (Zegers-Hochschild *et al*., 2022). Only 98 posts were retrieved. Most were authored by HCPs who used technical terms such as oncofertility, fertility preservation, and oocyte freezing almost exclusively. Technical terms may further impair patient comprehension of the subject, poorly explained on American fertility clinics’ websites (Mujumdar *et al*., 2022). We tried but failed to find other published studies to compare our results. As cancer rates are expected to increase in the coming years and women keep postponing pregnancy, the need for oncological fertility preservation should increase (Sehring *et al*., 2021).

The availability of clearly written, evidence-based information on social media will be crucial for individuals undergoing cancer treatment and their families. Social media platforms such as Instagram are an important means of spreading evidence-based information on oncofertility and fertility preservation and increasing awareness among laypeople and healthcare professionals.

This study included only Brazilian Portuguese Instagram accounts and excluded commercial posts and videos. Our analysis covered Brazil and followed the framework defined in previous studies (Perone *et al*., 2020; Peyser *et al*., 2021), with the primary aim of mapping the Brazilian Instagram fertility landscape. Although not discussed in this study, content quality analysis is a significant problem that cannot be overlooked in future studies to strengthen the dissemination of accurate information to patients. Many people follow the advice of “digital influencers.” Reproductive medicine specialists should ensure a solid online presence to counteract misinformation and obtain information about ideas and beliefs shared online that might affect optimal patient care and education (Blakemore *et al*., 2020; Metzler *et al*., 2022). Tools to make such evaluations must be developed to allow the proper analysis of this issue in the future.

In conclusion, our study shows that both healthcare professionals and laypeople share fertility-related posts on fertility on Instagram using various hashtags. Educational posts are the majority, followed by emotional and social support. Healthcare professionals concentrate on education and use more technical hashtags, whereas laypeople focus on emotional aspects. Instagram may play an essential role in educating and providing emotional support to people facing fertility issues. As Brazilians increasingly resort to Instagram to learn about various health topics, understanding who is behind the fertility-related posts may help reshape this landscape and disseminate good quality information. This is vital as access to proper medical evaluation and treatments is a problem in Brazil, and social media may be the sole source available to many. Further studies should concentrate on the quality of information provided by laypeople and healthcare professionals.
